# Mitigating spatial hallucination in large language models for path planning via prompt engineering

**DOI:** 10.1038/s41598-025-93601-5

**Published:** 2025-03-14

**Authors:** Hongjie Zhang, Hourui Deng, Jie Ou, Chaosheng Feng

**Affiliations:** 1 College of Computer Science, Sichuan Normal University, Chengdu, 610101 China; 2 School of Information and Software Engineering, University of Electronic Science and Technology of China, Chengdu, 611731 China

**Keywords:** Computer science, Software

## Abstract

Spatial reasoning in Large Language Models (LLMs) serves as a foundation for embodied intelligence. However, even in simple maze environments, LLMs often struggle to plan correct paths due to hallucination issues. To address this, we propose **S2ERS**, an LLM-based technique that integrates entity and relation extraction with the on-policy reinforcement learning algorithm Sarsa for optimal path planning. We introduce three key improvements: (1) To tackle the hallucination of spatial, we extract a graph structure of entities and relations from the text-based maze description, aiding LLMs in accurately comprehending spatial relationships. (2) To prevent LLMs from getting trapped in dead ends due to context inconsistency hallucination by long-term reasoning, we insert the state-action value function Q into the prompts, guiding the LLM’s path planning. (3) To reduce the token consumption of LLMs, we utilize multi-step reasoning, dynamically inserting local Q-tables into the prompt to assist the LLM in outputting multiple steps of actions at once. Our comprehensive experimental evaluation, conducted using closed-source LLMs ChatGPT 3.5, ERNIE-Bot 4.0 and open-source LLM ChatGLM-6B, demonstrates that **S2ERS** significantly mitigates the spatial hallucination issues in LLMs, and improves the success rate and optimal rate by approximately 29% and 19%, respectively, in comparison to the SOTA CoT methods.

## Introduction

Large Language Models makes remarkable breakthroughs in various fields such as social simulation^1^, natural science^2^, and education^3^. As LLMs are entering the era of embodied intelligence^4^, spatial reasoning becomes a fundamental aspect. Spatial reasoning is crucial for embodied agents to navigate and interact with their environment effectively. However, even in simple maze scenarios, LLMs often struggle to plan correct paths due to the hallucinations problem^5^. This issue significantly hinders the further development and practical application of LLMs in embodied intelligence.

To address the aforementioned challenges, researchers explore and improve the reasoning capabilities of LLM, primarily through instruction fine-tuning, extending the Chain-of-Thought (CoT)^6^, and integrating other advanced search algorithms. In terms of instruction fine-tuning, it tackles the hallucination by requiring a batch of manually labeled data, and fine-tuning the model, which consumes a lot of computational effort. For CoT, researchers extend it into a tree structure, turning it into Tree-of-Thought (ToT)^7^, and even into a graph structure, turning it into Graph-of-Thought (GoT)^8^. In addition, self-consistency CoT (CoT-SC) can be achieved through multiple reasoning paths to improve the reasoning accuracy^9^. Besides, there are also interaction-based methods, such as ReAct^10^ and Reflexion^11^. In terms of integrating advanced search algorithms, researchers propose algorithms that combine LLM with reinforcement learning (RL)^12–14^. For example, Rememberer uses a Q-table to assist LLM in exploring the space and avoiding the context inconsistency hallucination by long-term reasoning^15^. On the other hand, LLM also act as a reward function to guide the RL training^16^. Although the LLM planner based on prompts and advanced search algorithms can mitigate hallucination during long-term reasoning, yet hallucination still persist in understanding spatial coordinates^5^. Additionally, it necessitates a lot of tokens to facilitate interaction between the LLM and the maze environment, making it a costly approach.

**Fig. 1:**
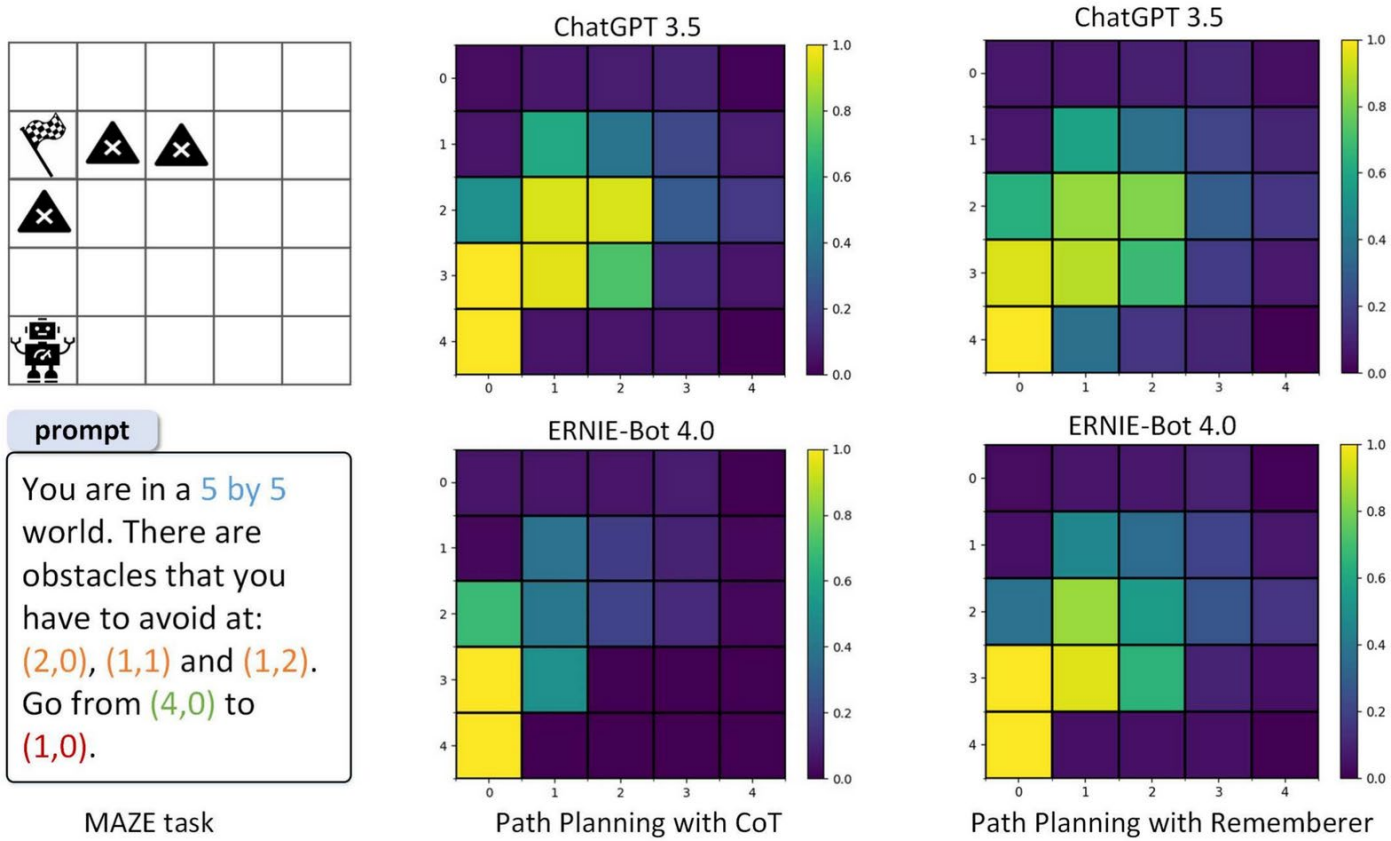
This is a path planning task in MAZE, which shows the spatial hallucination of LLM. Based on ChatGPT 3.5 and ERNIE-Bot 4.0, we use the Chain of Thought (CoT) and Rememberer algorithms for path planning. The color of heatmaps at each position indicates the number of times that position has been visited after multiple uses of CoT and Rememberer. In particular, in order to successfully reach the target, LLM often regards obstacle *(1,1)* as no obstacle.

We use a 2D MAZE as the target task of path planning to illustrate the spatial hallucination of LLM. Figure 1 displays a $5\times 5$ maze containing a starting point, an ending point, and obstacles, which we refer to as MAZE using natural language. Figure 1 also presents the heatmaps of the search for the shortest path using the LLM with the CoT and Rememberer algorithms, respectively. We found that CoT and Rememberer often got stuck near obstacles and could not reach the destination. Based on the analysis of the responses returned by LLM, we discovered that the agent always imagined a non-existent path to cross obstacles and reach the destination in a straight line. This is a hallucination problem of LLM regarding spatial relationships. Moreover, LLM tend to plan paths in greedy because of the inconsistency hallucination by long-term reasoning^5^.

Inspired by the above observations, we propose an LLM-based path planner **S2ERS** that combines spatial information conversion into an **Entity Relationship (ER)** graph and the Sarsa algorithm. To address LLMs’ hallucination of spatial relationships, we extract locations from the MAZE description as entities and the accessibility between locations as relationships, thus forming a spatial relationship graph. Then, in order to solve the long-term reasoning hallucination problem, we integrate the state-action value function, namely the Q-table, into the LLM prompt to assist long-term reasoning and find the shortest path. In addition, to reduce the number of tokens required by LLM, we incorporate multi-step reasoning method. Specifically, we dynamically insert a local Q-table into the prompt, allowing LLM to perform multi-step Q-value lookups, thus enabling the output of multiple actions at once and avoiding repeated ask the LLM. We design a batch of 2D MAZEs and robotic navigation tasks to conduct extensive experiments. The results show that compared to advanced prompt engineering, our **S2ERS** has significant improvements in both success rate and optimal rate. In addition, we reduce the number of tokens in the algorithm through multi-step reasoning.

In brief, the contributions of this paper are as follows:

1. We introduce a novel approach of transforming spatial text into a spatial relationship graph, aiming to mitigate spatial hallucination in LLMs and enhance reasoning accuracy.

2. We employ the n-step Sarsa learning to the spatial relationship graph, mitigating the long-term reasoning hallucination of LLM. This approach improves the success rate and reduces the token costs.

3. We have conducted extensive experiments using closed-source LLMs ChatGPT 3.5, ERNIE-Bot 4.0 and open-source LLM ChatGLM-6B to validate the superiority of our algorithm in terms of success rate and optimal rate by mitigating spatial hallucination in LLM.

## Related work

### Chain of thought

In order to fully leverage the reasoning capabilities of LLMs, Wei et al. propose the chain-of-thought technique, which requires LLMs to perform step-by-step reasoning through prompts^6^. This not only improves the ability to complete tasks but also provides interpretability. As CoT cannot explore multiple reasoning paths, LLMs cannot solve complex problems. Wang et al. propose CoT-SC^9^, which requires LLMs to output multiple reasoning paths simultaneously, and then make strategies to find the most representative result from them, such as majority vote. Yao et al. propose a tree-structured thought process, ToT, which allows the model to make multiple reasoning paths and look ahead or backtrack when necessary to make global decisions^7^. Researchers from ETH Zurich propose a brand-new LLM thinking framework, GoT, the main advantage of which is modeling the information generated by LLM as a graph^8^. Xiao et al. propose a multi-agent reasoning framework, namely Chain-of-Experts (CoE), to coordinate multiple LLM agents to solve complex or challenging problems^17^. Furthermore, researchers propose the ReAct method, which imitates the way humans operate in the real world, because we can conduct verbal reasoning and take actions to obtain information^10,18^. Shinn et al. propose the Reflexion mechanism, which can identify errors and self-reason how to improve by introducing self-reflection steps^11^. However, various CoTs perform unsatisfactorily when facing spatial path planning problems, which stems from the spatial hallucination and biases towards local optimum of LLM. Yang et al. introduce SignEye, a new method using CoT and structured descriptions for traffic signs, eliminating the need for complex training and extensive difficult-to-obtain symbol and text data^19^. We both use space descriptions for route planning and navigation, ensuring information completeness and accuracy without redundancy or omissions. They also use a step-by-step reasoning approach for path planning, reducing errors and enhancing explainability. SignEye extracts spatial info from images, while this work focuses on spatial relationships from text. And SignEye is about current vehicle actions, while S2ERS is for overall path planning.

### LLMs with advanced search

Inspired by cognitive science, Zhang et al. propose a novel evolvable LLMs-based agent framework called Rememberer^15^. By equipping LLMs with long-term empirical memory, Rememberer can leverage experiences from past episodes, even for different task goals, which is superior to LLMs-based agents with fixed examples. Carta et al. propose GLAM, which fine-tunes the action layer of LLMs’ output through the Proximal Policy Optimization (PPO) algorithm^12^. In addition, Zhao et al. utilize the world knowledge of LLMs to assist Monte Carlo Tree Search (MCTS) and improve search efficiency, such as LLM-MCTS^13^. In addition, LLMs can also assist reinforcement learning. Du et al. propose the ELLM algorithm, which combines current observations in the Crafter environment through LLMs to provide practical goals and rewards for each step of decision-making, guiding the agent to carry out meaningful exploration^14^. Kwon et al. use LLM to design human-desired rewards (texts) for different tasks based on the current state (text) in reinforcement learning, and digitize them for agent learning and training, which improves the learning efficiency of the agent^16^. AI2 propose the SwiftSage agent framework^20^. They obtain a small model through imitation learning and then integrated it with LLMs. In this way, the small model can be fine-tuned with a large amount of data to equip it with knowledge related to the environment and tasks, and the large model can be invoked only when necessary for higher-order reasoning. However, due to LLMs’ hallucination about spatial relationships and inability to accurately understand spatial positions, the prior knowledge, rewards, or thoughts provided by LLMs may be biased. In addition, interactive reasoning consumes a large number of tokens, so how to reduce the number of tokens is also an important issue.

## Methodology

### Overview

**Fig. 2:**
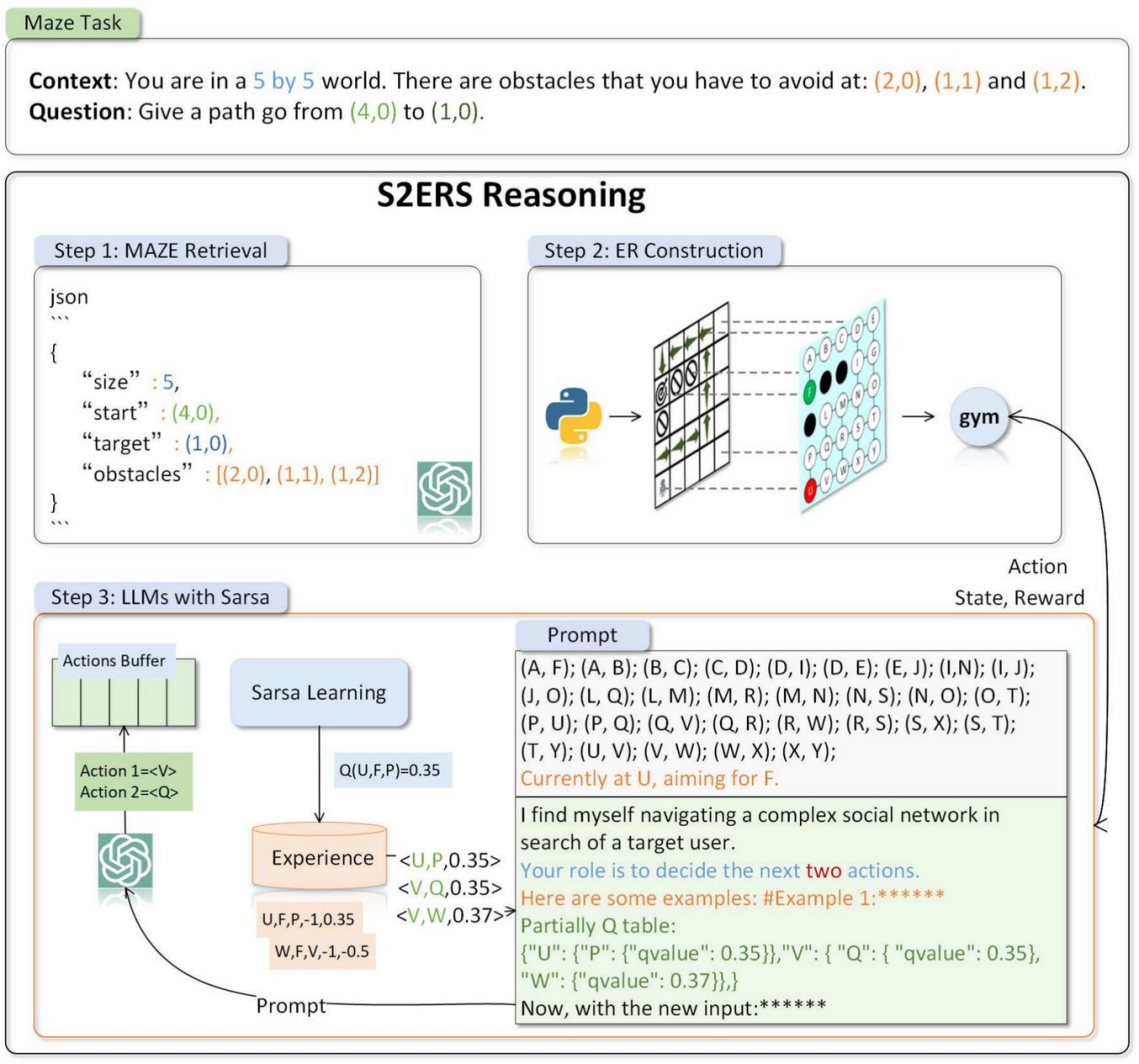
The overview of **S2ERS**. The above is a maze described in texts, and below are the three reasoning processes of **S2ERS**. LLM plan the shortest path based on the continuous interaction with the environment.

Figure 2 provides an overview of the **S2ERS** architecture introduced in this paper. Typically, we describe the MAZE task in natural language (texts), including the size, rules, starting point, goal, and non-passable areas of the maze. Extracting entities and relationships from LLM is challenging, and LLM may assume non-existent feasible areas due to its hallucination problem. Our **S2ERS** initially extracts structured information from text descriptions and constrains the output of LLM in JSON format, which enables the construction of a standardized entity relationship network using *Python*. Next, we treat locations as nodes and adjacent reachability as relationships, thereby constructing an entity relationship network in *Python*. Subsequently, we encapsulate the MAZE into a Gym environment, update the environment state based on LLM’s actions, and return rewards. Finally, we integrate the ReAct framework, which incorporates a local Q-table in prompt for path planning in the MAZE, preventing them from getting stuck due to long-term reasoning hallucination. To minimize the number of tokens used by LLM, we allow LLM to output multiple actions in a single planning step and utilize an **Action Queue** for interaction with the environment.

### Entity relationship construction

#### Entity extraction

In our MAZE, each position is an entity with a specific type, such as *(4,0)* being of type **start** and *(2,0)* being of type **obstacle**. We formalize entities as $E={(s_i,t_i)}_{i=1}^N$, where $s_i$ represents coordinates, $t_i$ represents types and *N* is the number of coordinates. Due to the difficulty of automatically extracting entities from texts using rule-based methods, we rely on LLM’s comprehension for entity extraction. Moreover, we observe that LLM, when dealing with numerical coordinates, still struggles with spatial hallucination, assuming the existence of non-existent paths. To address this, we convert numerical entities into letters, names, or a combination thereof, ensuring a lack of similarity between entities. We implement a direct one-to-one mapping using *Python*, for instance, mapping *(0,0)* to the letter *A*.

**Fig. 3:**
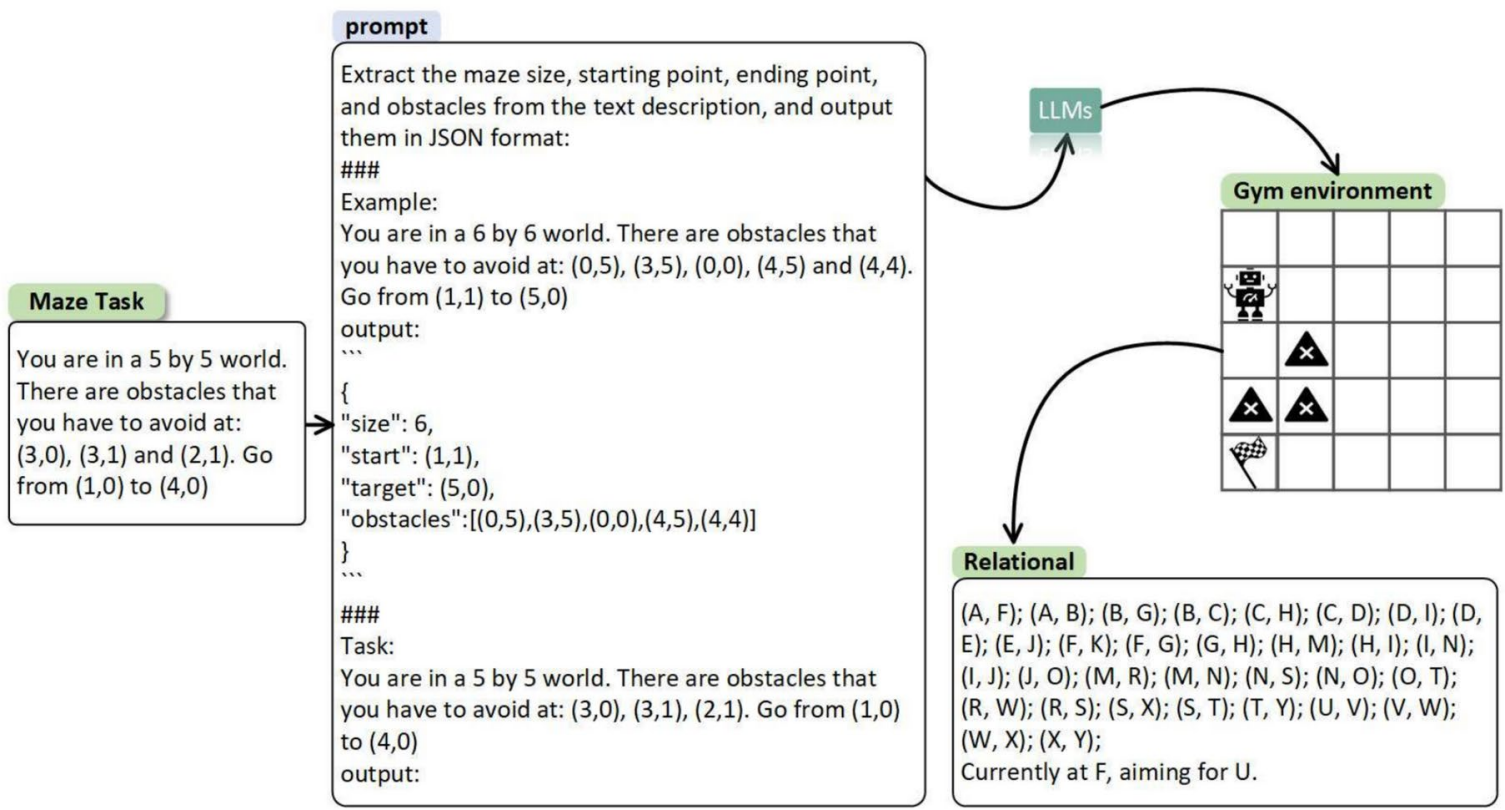
The process of converting MAZE description to ER diagram involves LLM entity extraction, *Python* entity naming, and relationship identification.

#### Relationship extraction

In the MAZE, the explicit relationship that can be directly extracted is the neighbor reachability. If two adjacent coordinates are reachable, then there is a relationship between these two positions, such as *(0,0)* and *(0,1)*. Otherwise, there is no relationship between them, such as *(0,1)* and *(1,1)*. Analogous to social networks, if two people know each other, there is a relationship between them. We only extract this explicit relationship, while complex implicit relationships are the targets that need to be reasoned by LLM. For example, *A* knows *B*, *B* knows *C*, which are explicit relationships. However, *A* can know *C* through *B*, which is an implicit relationship. We utilize the reasoning ability of LLM to identify such relationships. In addition, in order to save LLM tokens, we use the symbol (*A*, *B*) to indicate that there is a relationship between entity *A* and entity *B*, and they are mutually accessible. In MAZE, we build the relationship between nodes and all reachable neighbors through *Python* code.

Based on the above-mentioned entity extraction and relation recognition, we construct an ER graph for MAZE. This process is visualized in Fig. 3, where the MAZE is described in texts. We extract entities and their respective categories from the description, and then utilize *Python* code to map the coordinates to letters or strings. Ultimately, we establish accessibility relations to generate the final ER description, which served as the basis for subsequent path planning by LLM.

### LLM with Sarsa

LLM tends to fall into local solutions during the MAZE path planning process, due to the hallucination caused by LLM’s long-term reasoning. We found that in most cases, when LLM tries multiple times and finds that it cannot reach the target point, it firmly believes that there is no reachable path here and cannot proceed to the next step of reasoning. We use the Q-table in RL as the historical experience of LLM, and update the Q-table through continuous trial and error. LLM uses the Q-table to assist reasoning. We dynamically insert part of the Q-table into the prompt, thus alleviating the hallucination problem of LLM during long-term reasoning.

**Fig. 4:**
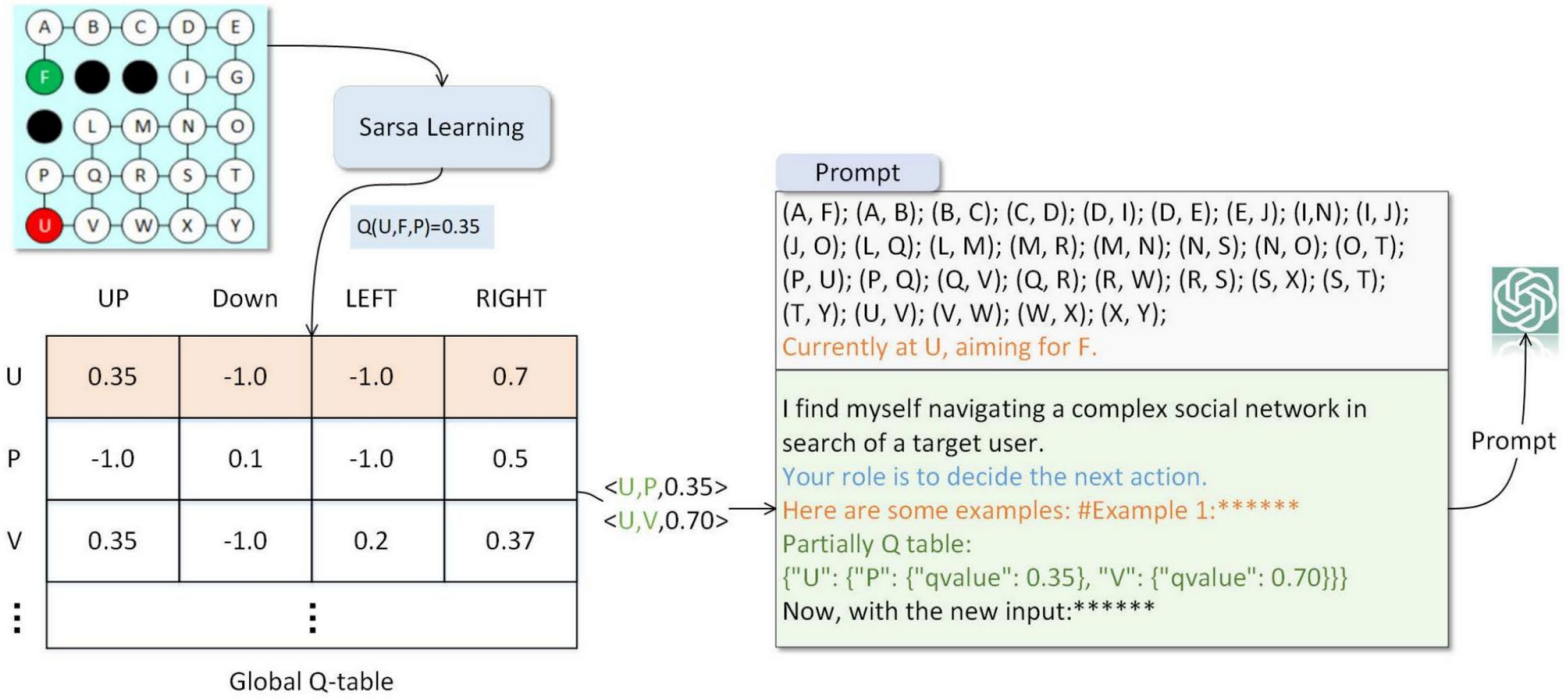
The process of embedding partially Q-table into the prompt. The global Q-table is updated by Sarsa, The Q-value of the current node is embedded into the prompt in **JSON** format.

In our MAZE task, there are two states: $s_t^g$ and $s_t^c$. $s_t^g$ includes the ER diagram, the starting point, the ending point, and the current node at time step *t*. LLM makes decisions based on $s_t^g$. Meanwhile, $s_t^c$ only includes the current node at time step *t* and is used to calculate the Q-table. The action choices for LLM are $A(i)=\{j|j\in ER(i)\}$, which are the neighbor nodes that are directly connected to the current node *i*. LLM picks a node *j* from *A*(*i*) as its action, and then the MAZE changes the current node *i* to the new node *j*. After LLM takes an action, the MAZE gives a reward $r_t$ for that step *t*. LLM gets a reward of $-1$ for each step it takes, which helps it find the shortest path. If LLM takes more detours, the reward gets lower. When LLM reaches the end point, it gets a reward of 30, which helps it reach the goal.

The traditional Sarsa algorithm uses an $\epsilon$-greedy policy to choose actions. It picks random actions with a chance of $\epsilon$ and the best action (the one with the highest Q-value) with a chance of $(1-\epsilon )$. But choosing random actions can make the program visit many states that are not useful, which wastes time. We changed this to use LLM instead, using their knowledge to explore more efficiently. Our way of choosing actions is shown in Eq (1), where $\epsilon$ is a preset exploration probability that gradually decreases as training progresses. *p* is the random value, and $LLM(s_t^g, Q\_table)$ puts part of the Q-table into the prompt to help it think better.

1 $$\begin{aligned} a_t = {\left\{ \begin{array}{ll} argmax_aQ(s_t^c, a) & if\quad p < 1-\epsilon \\ LLM(s_t^g, Q\_table) & otherwise \end{array}\right. } \end{aligned}$$

Figure 4 shows the process of adding Q-table. We construct a global Q-table, which contains all state-action pairs. We search the Q-values of different actions for the current node from the global Q-table, and write them into the prompt in **JSON** format to assist LLM in long-term reasoning.

### N-step reasoning

LLM just gives one action at a time, which wastes lots of tokens. This happens when examples or maze descriptions are repeated in prompt. We suggest using an **N-step** reasoning method, which lets LLM think ahead and give the next *N* actions at once. These actions are then put into an **Action Queue**. The Gym environment executes these actions one-by-one and gives rewards for each. We use the world-model of LLM like in model-based RL (MBRL^21^) to help predict what will happen next.

**Fig. 5:**
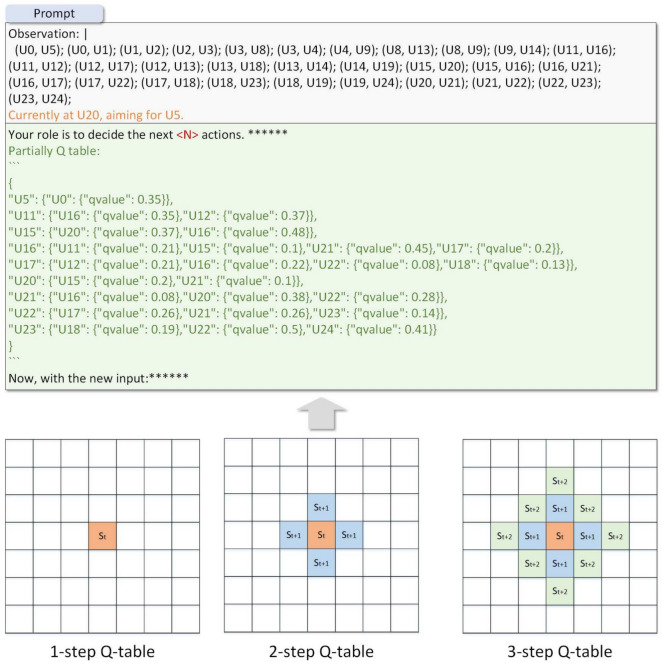
The partial Q-table in the prompt when $N\in (1,2,3)$. The current state is $s_t$, and the next state cover the 4 positions $s_{t+1}$.

In order to select the next action, LLM need to imagine the changes that will occur in the environment after executing action. We present a complete Entity-Relationship graph in the prompt, allowing LLM to predict the next state based on the action. In addition, when LLM arrive at a new position, their corresponding Q-value should also be re-obtained for subsequent reasoning. Placing the global Q-table in the prompt would waste a large number of tokens, and as the MAZE increases, the number of tokens used by the Q-table increases linearly. In **S2ERS**, we put the partial Q-table within the **N-step** range into the prompt, making it independent of the MAZE size. Figure 5 shows the partial Q-table in the prompt when $N\in (1,2,3)$. This **JSON** only contains Q-values within **N** steps of the current position.

## Experiments

### Experiment settings

To verify the effectiveness of **S2ERS**, we design various scales of MAZE and conduct experiments based on closed-source LLMs **ChatGPT 3.5**, **ERNIE-Bot 4.0** and open-source LLM **ChatGLM-6B**. Specifically, we design 30 MAZEs of $5\times 5$, 20 MAZEs of $7\times 7$, 10 MAZEs of $10\times 10$, 3 MAZEs of $50\times 50$, 3 MAZEs of $100\times 100$. For the convenience of MAZE research, we release all MAZE texts to the open source code. Furthermore, We extended the MAZE task into a **robotic navigation** task. Specifically, we designed a new environment that includes 2 batteries, a key, a door, and a goal. The rules are: the robot must collect all the batteries and the key before opening the door, then open the door and reach the goal, while also taking the shortest possible path. This paper uses Python 3.10 and Gym 0.24.0 for experimentation.

The baseline **Prompt Engineering** methods are as follows: **Naive**, **CoT**^18^, **ToT**^18^, **ReAct**^18^, **Rememberer**^15^.

The evaluation indicators we use include success rate and optimal rate. Due to the randomness of LLM reasoning, we conduct 10 times experiments on each maze to ensure the reliability of the experimental results.

1. Success Rate(%). The ratio that LLM successfully reach the target to the total number of runs. This metric only emphasizes the ability to reach the target and does not require the shortest path. The formal definition of this metric is shown in Eq. (2), where $\mathbb {N}_{suc}$ represents the number of successful attempts and $\mathbb {N}_{all}$ represents the total number of runs.
  2 $$\begin{aligned} SR = \frac{\mathbb {N}_{suc}}{\mathbb {N}_{all}} \end{aligned}$$
2. Optimal rate(%). The proportion of getting the shortest path in successful cases, which is shown in Eq. (3), where the $\mathbb {N}_{opt}$ represents the number of optimal cases. Specifically, there can be multiple shortest paths in a MAZE, but the length of the shortest path is unique. As long as the length of the resulting path reaches the minimum value.
  3 $$\begin{aligned} OR = \frac{\mathbb {N}_{opt}}{\mathbb {N}_{suc}} \end{aligned}$$

### Main results

**Table 1:** Experimental results on closed-source LLMs **ChatGPT 3.5**, **ERNIE-Bot 4.0** and open-source LLM **ChatGLM-6B**. Where the **Rememberer** and **S2ERS** are trained for 30 epochs. The best results are highlighted by bold and the best baseline models are marked by italics. The score in each cell is represented as “ChatGLM-6B[ChatGPT score, ERNIE-Bot score]”.

Method	$5\times 5$		$7\times 7$		$10\times 10$	
	SR (%)	OR (%)	SR (%)	OR (%)	SR (%)	OR (%)
Naive	9.2 [11.0,11.4]	9.5 [11.5,10.8]	8.5 [10.6,10.3]	9.3 [13.0,12.5]	7.4 [9.0,9.1]	8.5 [9.2,8.9]
CoT	12.5 [15.0,15.0]	13.5 [15.4,14.5]	11.1 [14.2,14.9]	12.8 [14.2,13.5]	8.9 [10.3,10.5]	9.7 [11.7,10.1]
ToT	13.7 [16.8,17.1]	14.3 [14.6,13.8]	12.0 [16.5,16.6]	13.7 [14.4,13.1]	9.0 [10.0,10.3]	11.1 [12.5,12.9]
ReAct	15.0 [17.6,17.4]	15.8 [23.5,22.8]	14.3 [17.4,16.1]	15.0 [21.6,21.6]	12.6 [14.8,15.4]	14.4 [21.3,20.7]
*Rememberer*	*37.6 [44.7,45.1]*	*46.4 [52.6,50.8]*	*34.8 [37.4,44.2]*	*37.8 [46.4,40.2]*	*32.5 [32.6,34.8]*	*30.5 [36.6,35.7]*
$S2ERS(1\ step)$	65.4 [72.4,73.7]	59.6 [67.1,65.6]	59.5 [64.7,64.6]	52.5 [59.2,57.4]	51.9 [55.1,53.5]	46.7 [53.7,51.7]
$S2ERS(2\ step)$	67.7 [75.1,76.2]	62.9 [69.6,67.7]	61.2 [66.2,67.2]	54.2 [63.7,59.8]	53.5 [56.4,56.3]	48.4 [55.9,52.9]
$S2ERS(3\ step)$	**68.7 [76.5,77.1]**	**64.0 [71.4,68.4]**	**61.9 [67.5,69.9]**	**55.4 [66.7,63.4]**	**54.6 [58.2,59.7]**	**50.0 [57.4,54.9]**

**Fig. 6:**
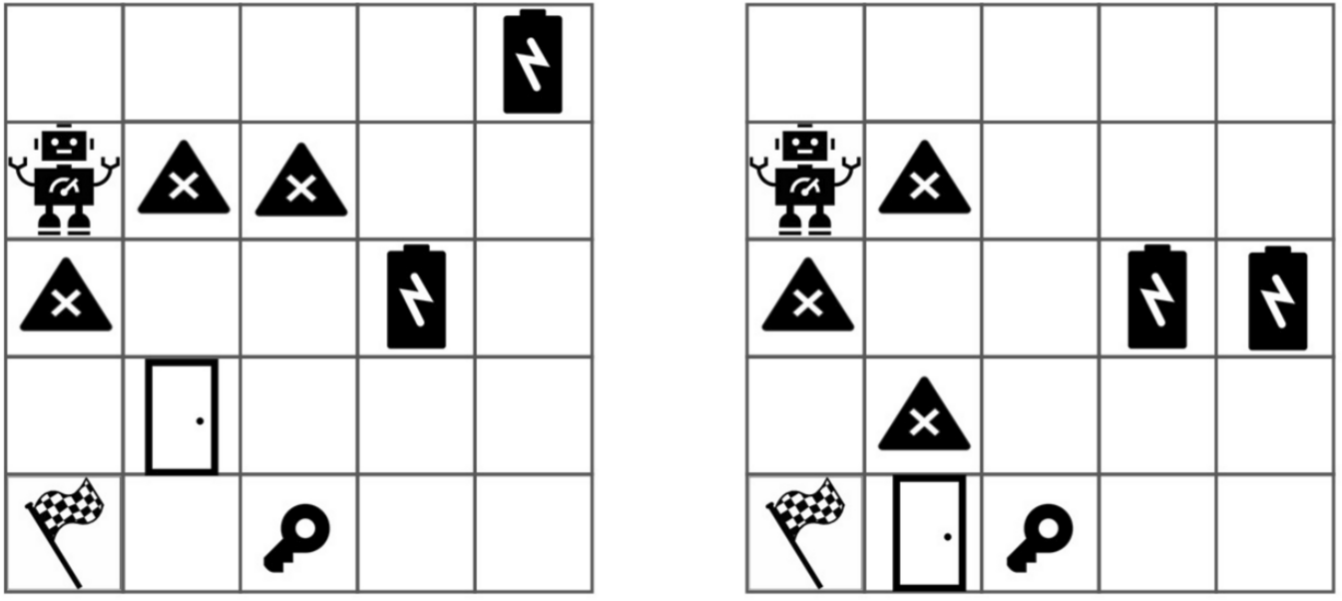
Robotic navigation tasks. The rules are: the robot must collect all the batteries and the key before opening the door, then open the door and reach the goal, while also taking the shortest possible path.

Table 1 presents the experimental results of various algorithms on different MAZEs based on closed-source LLMs ChatGPT 3.5, ERNIE-Bot 4.0 and open-source LLM ChatGLM-6B. The results indicate that our **S2ERS** improves the success rate by 29.2% and the optimal rate by 20.0% compared to the state-of-the-art **Rememberer** algorithm with ChatGPT 3.5. It improves the success rate by 28.9% and the optimal rate by 18.1% with ERNIE-Bot 4.0. And it improves the success rate by 26.8% and the optimal rate by 18.2% with ChatGLM-6B. We notice that the **Rememberer** algorithm, which combines the prior knowledge of LLM and the search capabilities of RL, significantly improves both sample utilization and policy quality, making it the optimal choice among the baselines. Even with advanced chain-of-thought techniques, LLM are unable to complete path planning in MAZE. We enlarge the size of the maze to $50\times 50$, $100\times 100$ and more obstacles, in order to truly understand the effectiveness and validity of proposed method. Experimental results in Table 2 show that on larger MAZEs, S2ERS still achieves the best performance. Table 3 shows the success rates and optimal rates of different algorithms in robot navigation tasks (shown in Fig. 6). The rules are: the robot must collect all the batteries and the key before opening the door, then open the door and reach the goal, while also taking the shortest possible path. Experimental results show that our S2ERS has achieved the best results in both success rate and optimal rate.

**Table 2:** Experimental results on larger MAZE. Where the **Rememberer** and **S2ERS** are trained for 10 epochs in 3 mazes. The best results are highlighted by bold and the best baseline models are marked by italics. The score in each cell is represented as “$SR (\%)$ score [$OR (\%)$]”. Particularly, traditional prompt engineer (Naive, CoT, ToT and ReAct) almost cannot succeed in such a large MAZE.

Method	$50\times 50$			$100\times 100$		
	ChatGPT 3.5	ERNIE-Bot	ChatGLM-6B	ChatGPT 3.5	ERNIE-Bot	ChatGLM-6B
*Rememberer*	*33.3 [50.0]*	*33.3 [40.0]*	*26.7 [37.5]*	*26.7 [50.0]*	*26.7 [50.0]*	*20.0 [50.0]*
$S2ERS(1\ step)$	56.7 [58.8]	60.0 [50.0]	50.0 [53.3]	46.7 [50.0]	50.0 [46.7]	40.0 [41.7]
$S2ERS(2\ step)$	60.0 [55.6]	60.0 [55.6]	50.0 [60.0]	46.7 [57.1]	53.3 [50.0]	43.3 [46.2]
$S2ERS(3\ step)$	**66.7 [70.0]**	**66.7 [65.0]**	**53.3 [56.2]**	**50.0 [66.7]**	**53.3 [62.5]**	**43.3 [46.2]**

In addition, as the number of prediction steps increases, the success rate and optimization rate of our **S2ERS** algorithm improve significantly. We believe that this is similar to n-step RL, where the use of n-step Q-value estimation balances bias and variance, making it towards the unbiased estimation. In addition, Table 4 demonstrates that as the number of reasoning steps *n* increases, the number of tokens used in the S2ERS algorithm gradually decreases. This is due to the reduction of repeated ER texts, few-shot cases, etc.

There are also some mature technologies for token compression. We applied the **LLMLingua**^22^ technology to S2ERS to further compress the number of tokens and reduce inference costs. The experimental results are shown in Table 5. Using LLMLingua can further compress the number of tokens by about 43%, but there is a semantic misunderstanding in the prompt, resulting in a decrease in both success rate and optimal rate.

**Table 3:** Experimental results on robot navigation tasks. Where the **Rememberer** and **S2ERS** are trained for 30 epochs. The best results are highlighted by bold and the best baseline models are marked by italics. The score in each cell is represented as “$SR (\%)$ score [$OR (\%)$].” Environment: A maze of 5 * 5, with two batteries, a key, a door and a target.

Method	ChatGPT 3.5	ERNIE-Bot	ChatGLM-6B
Naive	11.0 [4.5]	11.4 [4.0]	9.2 [3.5]
CoT	15.0 [5.7]	15.0 [5.5]	12.5 [4.5]
ToT	16.4 [5.6]	17.0 [4.5]	12.3 [4.5]
ReAct	17.7 [10.5]	16.5 [9.8]	15.0 [7.8]
*Rememberer*	*46.4 [32.6]*	*42.1 [30.3]*	*37.9 [28.6]*
$S2ERS(1\ step)$	71.0 [42.5]	73.4 [41.6]	64.7 [39.5]
$S2ERS(2\ step)$	73.5 [47.0]	74.2 [45.7]	66.7 [42.0]
$S2ERS(3\ step)$	**75.3 [50.5]**	**76.8 [48.8]**	**66.7 [45.5]**

**Table 4:** Token consumption based on ChatGPT 3.5 and ERNIE-Bot 4.0. Each cell is represented as “ChatGPT[ERNIE-Bot]”.

Method	$5\times 5$	$7\times 7$	$10\times 10$
$S2ERS(1\ step)$	274623 [278055]	348996 [352030]	1438164 [1448262]
$S2ERS(2\ step)$	143630 [153525]	219570 [214488]	973660 [965508]
$S2ERS(3\ step)$	116006 [118236]	158391 [161519]	751299 [758613]

**Table 5:** The compression rate of **LLMLingua** is approximately 43%, which resulting in a decrease in both success rate and optimality rate in MAZE $100\times 100$.

LLMs	Methods	Org tokens	Compress tokens	Org $SR(\%) [OR(\%)]$	Compress $SR(\%) [OR(\%)]$
ChatGPT 3.5	$S2ERS(1\ step)$	14390472	6177195	46.7 [50.0]	26.7 [50.0]
	$S2ERS(2\ step)$	9738653	4191709	46.7 [57.1]	26.7 [62.5]
	$S2ERS(3\ step)$	7518269	3239426	50.0 [66.7]	33.3 [60.0]
ERNIE-Bot	$S2ERS (1\ step)$	14480451	6234835	50.0 [46.7]	26.7 [37.5]
	$S2ERS(2\ step)$	9649215	4152758	53.3 [50.0]	30.0 [33.3]
	$S2ERS(3\ step)$	7585293	3260080	53.3 [62.5]	33.3 [50.0]
ChatGLM-6B	$S2ERS (1\ step)$	20132092	8648719	40.0 [41.7]	20.0 [33.3]
	$S2ERS(2\ step)$	13521289	5813298	43.3 [46.2]	20.0 [50.0]
	$S2ERS(3\ step)$	10522810	4516260	43.3 [46.2]	23.3 [42.9]

### Ablation study

Our **S2ERS** includes two main modules, the ER construction module and the n-step Sarsa module. In this section, we verify the effectiveness of the ER construction module, which is shown in Table 6. By explicitly identifying entities and relationships, the ER module can significantly reduce the spatial hallucination problem in LLM. We compare ChatGPT 3.5 and ERNIE-Bot 4.0, and find that whether the CoT or LLM+RL (Rememberer), the success rate and optimization rate both increase significantly after adding the ER module. We believe that explicitly adding entities and relationships to the prompt in the transformer model of LLM can fully utilize the self-attention mechanism. The reasoning process becomes continuously focusing on relevant entities in the encoder, which will reduce **hallucinations** and make the reasoning more reasonable. Even with a simple **Naive** prompt, the ER module can double the success rate and optimal rate. This indicates that ER is more friendly to LLM in understanding relationships and can fully unleash its potential.

**Table 6:** Enhancement of various prompt engineering with the aid of the ER module. The score in each cell is represented as “ChatGPT score [ERNIE-Bot score]”.

Method	$5\times 5$		$7\times 7$		$10\times 10$	
	SR(%)	OR(%)	SR(%)	OR(%)	SR(%)	OR(%)
Naive	11.0 [11.4]	11.5 [10.8]	10.6 [10.3]	13.0 [12.5]	9.0 [9.1]	9.2 [8.9]
Naive+ER	24.4 [25.3]	28.7 [27.1]	13.1 [13.3]	24.7 [23.9]	9.5 [10.1]	20.5 [18.7]
CoT	15.0 [15.0]	15.4 [14.5]	14.2 [14.9]	14.2 [13.5]	10.3 [10.5]	11.7 [10.1]
CoT+ER	31.6 [30.9]	34.1 [33.5]	20.4 [20.9]	24.5 [23.1]	12.8 [13.5]	21.3 [19.4]
ToT	16.8 [17.1]	14.6 [13.8]	16.5 [16.6]	14.4 [13.1]	10.0 [10.3]	12.5 [12.9]
ToT+ER	32.9 [33.4]	39.7 [38.8]	23.5 [24.6]	28.0 [27.5]	14.0 [14.5]	22.2 [20.9]
ReAct	17.6 [17.4]	23.5 [22.8]	17.4 [16.1]	21.6 [21.6]	14.8 [15.4]	21.3 [20.7]
ReAct+ER	35.3 [35.7]	42.0 [40.8]	26.4 [27.6]	31.4 [30.3]	18.2 [19.7]	25.0 [23.6]
Rememberer	44.7 [45.1]	52.6 [50.8]	37.4 [40.2]	46.4 [44.2]	32.6 [34.8]	36.6 [35.7]
Rememberer+ER	61.7 [61.9]	62.1 [59.6]	54.4 [55.6]	48.5[47.9]	46.2[47.5]	42.5[41.1]

We validate the improvement brought by the ER module using ChatGPT 3.5 on the Rememberer algorithm. The following text describes a simple MAZE environment and plans the shortest path to the target through the **Rememberer** algorithm.

***Prompt***:

*I’m now in a square maze and need to find the exit (target location). You need to help me make decisions about my next action. In the maze, my available actions are:*

*turn right -> (x, y + 1)*

*turn up -> (x + 1, y)*

*turn left -> (x, y - 1)*

*turn down -> (x - 1, y)*

*Example:*

*You are in a 5 by 5 world. There are obstacles that you have to avoid at: (2,0) (1,1) and (1,2) Go from (0,0) to (1,0).*

*Encouraged:*

*turn up -> 10 Get into the target*

*Diacourage:*

*turn down -> -0.1 Keep me away from the target location.*

*Now the new input:*

*You are in a 5 by 5 world. There are obstacles that you have to avoid at: (2,0) (1,1) and (1,2) Go from (1,0) to (1,3).*

***Response:***

*Given your starting position at (1,0) and the target position at (4,0), let’s try to navigate through the maze. the optimal step is:*

Encouraged:

- *turn up -> 0.2 (This step moves you towards the target while avoiding the nearby obstacles.) *

*Discouraged:*

- *turn down -> -0.1 (To avoid obstacles) *

*Based on the provided information, turning up seems like the most direct path to reach the target while avoiding obstacles.*

From the feedback of the LLM, we found that through a series of inferences, “turn up” was finally chosen, which led to entering the obstacle and failing the task. Then, we convert the MAZE described in text into an ER graph and use **Rememberer** for path planning. The corresponding prompt and response are shown below:

***Prompt***:

*I find myself navigating a complex social network in search of a target user. Help me find a path from the current node to the target node.*

*Now, with the new input:*

*(A, F); (A, B); (B, C); (C, D); (D, I); (D, E); (E, J); (I,N); (I, J); (J, O); (L, Q); (L, M); (M, R); (M, N); (N, S); (N, O); (O, T); (P, U); (P, Q); (Q, V); (Q, R); (R, W); (R, S); (S, X); (S, T); (T, Y); (U, V); (V, W); (W, X); (X, Y);*

*Currently at F, aiming for I.*

Based on the response from the LLM, we found that the LLM is able to plan the correct path, with guaranteed logical reasoning and more concise tokens. Experiments on **Rememberer** verify that our ER module can indeed reduce spatial hallucination and enhance the reasoning ability of the LLM.

***Response:***

*To reach node I from node F, here’s a path:*

1. *F -> A (via (A, F)) *

2. *A -> B (via (A, B)) *

3. *B -> C (via (B, C)) *

4. *C -> D (via (C, D)) *

5. *D -> I (via (D, I))*

*So, the path from F to I is: F -> A -> B -> C -> D -> I.*

### Toy example

**Fig. 7:**
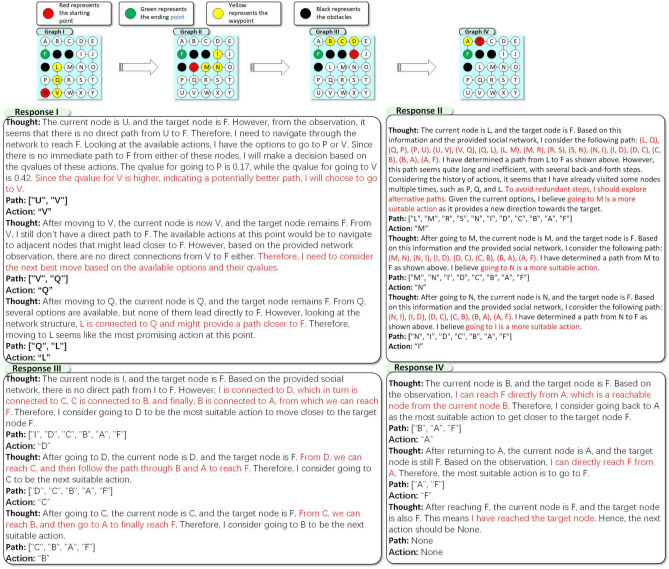
S2ERS with 3-step reasoning, which predicts 3 actions at each round.

In this section, we analyze the reasoning examples of **S2ERS** on MAZE, and delve deeper into the decision-making process from the responses of LLM. We conduct a comparative experiment based on ChatGPT 3.5. Figure 7 illustrates the reasoning process of our **S2ERS** algorithm on the MAZE task. Specifically, the LLM need to engage in step-by-step thinking, predict subsequent path sequences, and consider the Q-values for potential actions. Specifically, the following response shows the reasoning process with Q-values. From the LLMs’ response, we can see that it engages in thoughtful consideration and utilizes its own forward function for multi-step reasoning.

***Thought:*** *The current node is U, and the target node is F. However, from the observation, it seems that there is no direct path from U to F. Therefore, I need to navigate through the network to reach F. Looking at the available actions, I have the options to go to P or V. Since there is no immediate path to F from either of these nodes, I will make a decision based on the qvalues of these actions. The qvalue for going to P is 0.17, while the qvalue for going to V is 0.42. Since the qvalue for V is higher, indicating a potentially better path, I will choose to go to V.*

When making decisions, LLM no longer relies on long-term reasoning to judge the feasibility of a path, which can easily lead to the context inconsistency hallucination. Instead, the Q value already represents the likelihood of reaching the goal if a certain action is taken, eliminating the need for further reasoning.

**Fig. 8:**
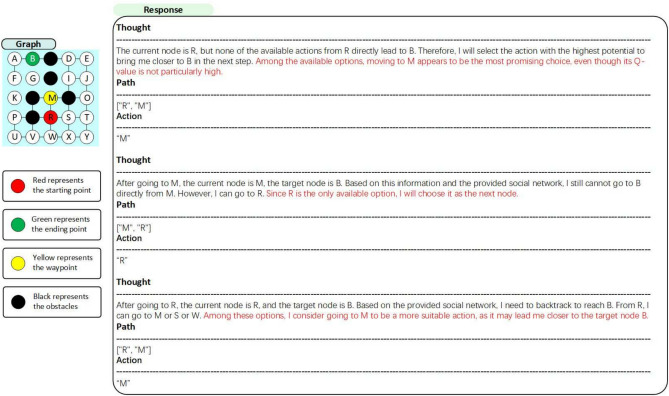
Failure case analysis. LLMs overly trust intuition and ignore the Q-table, leading to failure.

S2ERS does not have a 100% success rate. We have analyzed some of the typical errors, as shown in Figure 8. We found that in this MAZE, S2ERS always trusts its own incorrect intuition, even when the Q-table provides the optimal path. The thought like this:

***Thought1:*** *The current node is R, but none of the available actions from R directly lead to B. Therefore, I will select the action with the highest potential to bring me closer to B in the next step. Among the available options,* ***moving to M appears to be the most promising choice, even though its Q-value is not particularly high.***

**Thought2:** *After going to M, the current node is M, the target node is B. Based on this information and the provided social network, I still cannot go to B directly from M. However, I can go to R.* ***Since R is the only available option, I will choose it as the next node.***

In future work, we will further emphasize the role of the Q-table in the prompt to reduce the issue of hallucinations and prevent S2ERS from getting stuck wandering between R and S, thereby improving the success rate.

## Conclusion

This paper proposed a MAZE path planning framework called **S2ERS**. The framework consists of two main modules: the Entity-Relation Extraction Module and the n-step Policy Module. Firstly, the Entity-Relation Extraction Module extracts nodes and the connections from the text description of the MAZE to mitigate spatial hallucination. Secondly, to mitigate context inconsistency hallucination by long-term reasoning, the n-step Policy Module puts a partial Q-table in the prompt. Extensive experiments were conducted on closed-source LLMs ChatGPT 3.5, ERNIE-Bot 4.0 and open-source LLM ChatGLM-6B. We have conducted extensive experiments on various maze and robot navigation tasks. The results demonstrated that significant improvements in both success rate and optimal rate for our **S2ERS** algorithm. Additionally, transplanting the ER module to other baseline algorithms significantly enhances their performance. Our future research direction will focus on two aspects, using LLM to solve large-scale path planning problems, and reducing LLM reasoning time through a variety of technical.

## Supplementary Information

Supplementary Information.

## Data Availability

All data generated or analysed during this study are included in this published article and supplementary information files.

## Code availability

Source code is available at Code.
